# Aspirin-triggered resolvin D1 modulates pulmonary and neurological inflammation in an IL-22 knock-out organic dust exposure mouse model

**DOI:** 10.3389/fimmu.2024.1495581

**Published:** 2024-12-17

**Authors:** Alissa N. Threatt, Jade White, Nathan Klepper, Zachary Brier, Logan S. Dean, Ash Ibarra, Macallister Harris, Kaylee Jones, Maëlis J. L. Wahl, Melea Barahona, Emmanuel O. Oyewole, Morgan Pauly, Julie A. Moreno, Tara M. Nordgren

**Affiliations:** ^1^ Department of Environmental and Radiological Health Sciences, College of Veterinary Medicine and Biomedical Sciences, Colorado State University, Fort Collins, CO, United States; ^2^ Department of Biology, College of Natural Sciences, Colorado State University, Fort Collins, CO, United States; ^3^ Department of Animal Sciences, College of Agricultural Sciences, Colorado State University, Fort Collins, CO, United States; ^4^ Department of Biomedical Sciences, College of Veterinary Medicine and Biomedical Sciences, Colorado State University, Fort Collins, CO, United States; ^5^ Cell and Molecular Biology Graduate Program, Colorado State University, Fort Collins, CO, United States; ^6^ Department of Chemistry, College of Natural Sciences, Colorado State University, Fort Collins, CO, United States; ^7^ Experimental Pathology Facility, Department of Microbiology, Immunology, and Pathology, College of Veterinary Medicine and Biomedical Sciences, Colorado State University, Fort Collins, CO, United States; ^8^ Department of Biochemistry and Molecular Biology, College of Natural Sciences, Colorado State University, Fort Collins, CO, United States; ^9^ Brain Research Center, Colorado State University, Fort Collins, CO, United States

**Keywords:** agriculture dust, lung inflammation, neuroinflammation, omega-3 fatty acids, SPM, aspirin-triggered resolvin D1, AT-RvD1

## Abstract

Agriculture dust contains many organic immunogenic compounds, and organic dust exposure is strongly associated with the development of immune-mediated chronic pulmonary diseases such as chronic obstructive pulmonary disease (COPD). Chronic organic dust exposure from agriculture sources induces chronic lung inflammatory diseases and organic dust exposure has recently been linked to an increased risk of developing dementia. The cytokine interleukin-22 (IL-22) has been established as an important mediator in the resolution and repair of lung tissues. The omega-3 fatty acid metabolite aspirin-triggered Resolvin D1 (AT-RvD1) has shown efficacy in modulating the immune response in both pulmonary and neurological inflammation but has not been explored as a therapeutic in organic dust exposure-induced neuroinflammation. Investigating the link between IL-22 and AT-RvD1 may help in developing effective therapies for these immune-mediated diseases. We aimed to investigate the link between organic dust exposure and neuroinflammation, the role of IL-22 in the pulmonary and neurological immune response to organic dust exposure, and the immune-modulating therapeutic applications of AT-RvD1 in an IL-22 knock-out mouse model of organic dust exposure. C57BL/6J (WT) and IL-22 knock-out (KO) mice were repetitively exposed to aqueous agriculture organic dust extract (DE) 5 days per week for 3 weeks (15 total instillations) and treated with AT-RvD1 either once per week (3 total injections) or 5 times per week (15 total injections) for 3 weeks and allowed to recover for 3 days. We observed a significant pulmonary and neurological immune response to DE characterized by the development of inducible bronchus associated lymphoid tissue in the lung and gliosis in the frontal areas of the brain. We also observed that IL-22 knock-out increased pulmonary and neurological inflammation severity. Animals exposed to DE and treated with AT-RvD1 displayed reduced lung pathology severity and gliosis. Our data demonstrate that DE exposure contributes to neurological inflammation and that IL-22 is crucial to effective tissue repair processes. Our data further suggest that AT-RvD1 may have potential as a novel therapeutic for organic dust exposure-induced, immune-mediated pulmonary and neurological inflammation, improving outcomes of those with these diseases.

## Introduction

The Centers for Disease Control and Prevention ranked chronic lower respiratory diseases (CLRD) as a leading cause of death in the United States (US) with 147,367 deaths in 2023 (1). Chronic inflammatory lung diseases have also been linked to neuroinflammation and the development of neurodegenerative diseases such as Alzheimer’s (2–5). In 2023, 6.7 million people were reported as diagnosed and living with Alzheimer’s disease in the US, and estimates project that number to increase to 14 million people by 2060 (1, 6). CLRDs include chronic obstructive pulmonary disease (COPD), asthma, and allergies (7–9). Particulate matter (PM) exposure is a significant occupational hazard for agriculture workers, with organic dust exposure strongly linked to occupational-associated CLRDs (10–13). Organic dusts contain a variety of particulate matter (PM) sizes from 2.5 μm to 0.25 μm, microbes, and many immunogenic compounds such as microbial components, endotoxins, and metals that contribute to the pulmonary immune response (14, 15).

Our laboratory has extensively characterized the pulmonary immunological and pathological effects of organic dust exposure in a murine model of repetitive dust exposure, but the secondary neuroinflammatory effects of inhaled organic dust exposure have not been explored (10, 16–19). Inhaled PM exposure has been recently linked to an increased risk of developing dementia, with the greatest risk being associated with agricultural organic dust exposure over all other PM exposures (20). COPD patients have documented increases in depressive symptoms, confusion, memory loss, and mental functional decline as their disease progresses (21, 22). A study conducted by the CDC analyzed comorbidities of 8,094 patients in resident care facilities diagnosed with CLRDs including chronic bronchitis, emphysema, and COPD (23). This study found that 51.4% of patients with one more CLRD also presented with Alzheimer’s disease or dementia (p <0.001), 27.4% were diagnosed with depression (p=0.012), 7.7% were diagnosed with multiple sclerosis, Parkinson’s disease, or epilepsy (p= 0.020), and 11.7% presented with other mental disorders (p=0.007) (23). Overall, the authors found that 64.9% of patients with more CLRD experienced a mental or behavioral health disorder, and 11.6% experienced a nervous system disorder (23). Agriculture workers often experience high levels of mental disorders including anxiety (males: 22% females: 39%, total: 31%) and depression (males: 35%, females: 42%, total: 39%) (24). Additionally, air pollution exposure has been linked to neurological inflammation in murine models but has not been assessed in the specific context of agricultural organic dust exposure (3, 25, 26).

Both COPD and Alzheimer’s are incurable, progressive, and ultimately fatal diseases with severe symptoms that reduce patients’ quality of life during the progression of disease (25, 27–29). The search for therapies that combat these diseases is thus at the forefront of immunotoxicological research. Omega-3 fatty acid metabolites termed specialized pro-resolving mediators (SPMs), have been proposed as exogenous therapies for a variety of inflammatory diseases, suggesting they may be effective in reducing inflammation in a repetitive organic dust exposure model of pulmonary and neurological inflammation (30, 31). Aspirin-Triggered Resolvin D1 (AT-RvD1), a SPM, has demonstrated restorative functions in pulmonary and neuroinflammation models (32–35). Specifically, the aspirin-triggered 17(R)-RvD1 epimer has shown increased stability and pharmacological efficacy compared to its 17(S)-RvD1 epimer in murine models of acute lung injury and a chronic organic dust exposure-mediated pulmonary inflammation murine model (32, 36–38).

Interleukin-22 (IL-22), an interleukin-10 (IL-10)-family cytokine has been implicated in inflammation modulation, tissue repair, and antimicrobial defense (39). Murine models investigating the inflammatory consequence of IL-22 knock-out in infection, allergy, and organic dust exposure models have demonstrated increased disease severity in animals lacking IL-22 (40–45). We recently demonstrated that whole-body IL-22 knock-out mice exhibit increased pathology severity, cellular infiltrate counts, pro-inflammatory cytokine production, and altered tissue pathology following repetitive organic dust exposure (45). We aimed to investigate the link between attenuation of IL-22 and the tissue repair functions of AT-RvD1 in an IL-22 knock-out mouse model repetitively exposed to organic dust exposure.

We explored the link between inhaled organic dust exposure-induced pulmonary and neurological inflammation via a mouse model repetitively exposed to aqueous agriculture organic dust extract (DE). We also aimed to investigate the SPM AT-RvD1 as an exogenous therapy for pulmonary and neurological inflammation utilizing an IL-22 knock-out transgenic mouse model of severe pulmonary inflammation to explore its role in modulating lung-brain axis inflammation. To investigate our hypotheses, we evaluated the inflammatory response to organic dust exposure and the immune modulation of AT-RvD1 in an IL-22 knock-out model repetitively exposed to DE through evaluation of lung immune cell infiltrates, bronchoalveolar lavage fluid (BALF) and lung tissue cytokines, lung pathology, brain microglia quantification, and transcript evaluation in lung and brain tissue via RNAscope technology.

## Materials and methods

### Dust extract preparation

Dust was collected from hog confinement facilities in the Midwest, United States from surfaces at least 1 meter off the ground to represent the respirable fraction. Dust aliquots were stored at -20°C until use. Dust extracts were prepared as previously described (18, 46). Briefly, whole dust was combined with Hank’s balanced saline solution (HBSS) (HyClone Laboratories) at a ratio of 1 g dust to 10 mL HBSS on a magnetic stir plate for 1 hour at room temperature. The resulting mixture was centrifuged at 2500 rpm for 20 minutes at 4°C. The supernate was collected, while the pellet was discarded. Supernate was transferred to a new tube and centrifuged again at 2500 rpm for 20 minutes at 4°C. Supernate was collected and filtered through 0.22 μm syringe filters to produce 100% dust extract (DE) and stored at -20°C. The resulting extract contains predominantly gram-positive bacterial components, endotoxins, and trace metals (15). Complete analysis of dust extract can be found at Online Repository Methods at www.jacionline.org (15).

### Animal husbandry and care

All animal protocols were reviewed and approved by the Institutional Animal Care and Use Committee (Protocol Number 2887). C57BL/6J (WT) and whole-body IL-22 knock-out [C57BL/6-*Il22^tm1.1(icre)Stck^*/J] (KO) (Jackson Labs) mice aged 8-12 weeks were housed in the Colorado State University Painter Facility in a specific pathogen-free environment with free access to standard mouse feed and water. Three pairs of KO animals were purchased to establish a breeding colony of IL-22cre x IL-22cre that produced all experimental animals. Animals from the original breeding pairs and all offspring were genotyped through TransnetYX (Memphis, TN) genotyping PCR service to ensure accurate genotypes. WT mice were purchased directly from Jackson Laboratories, age and sex matched to KO mice. Purchased mice were acclimated for at least 7 days before performing any procedures.

### *In vivo* repetitive DE exposure and AT-RvD1 treatment model

**Figure 1:**
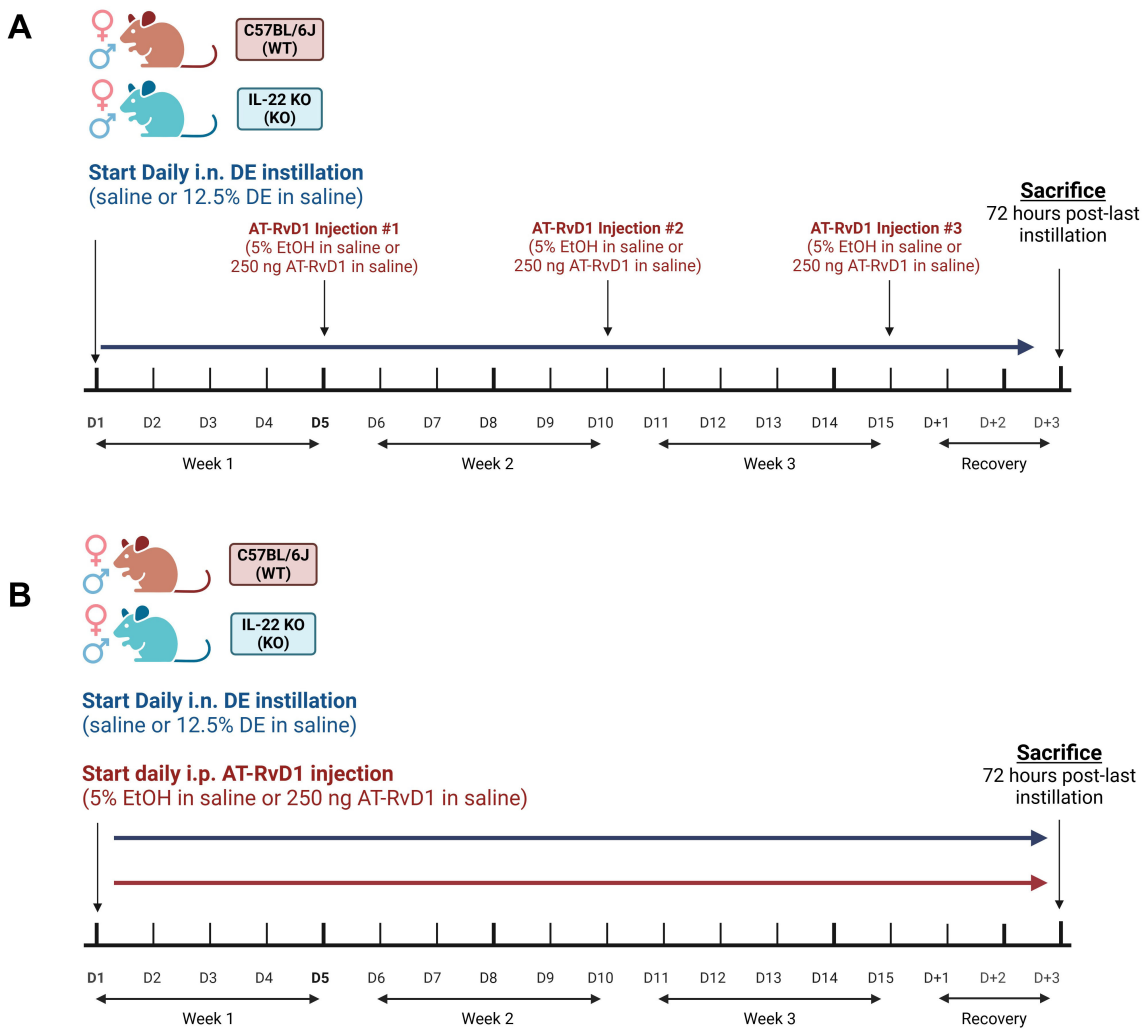
AT-RvD1 dosing strategies in WT and IL-22 KO mice. **(A)** once weekly AT-RvD1 dosing strategy timeline; WT and KO animals were instilled i.n. with 12.5% DE 5 days/week for 3 weeks and treated with 250 ng AT-RvD1 i.p. once/week for 3 weeks, **(B)** Once daily AT-RvD1 dosing strategy timeline; WT and KO animals were instilled i.n. with 12.5% DE 5 days/week for 3 weeks and treated with 250 ng AT-RvD1 i.p. 5 days/week for 3 weeks. Created with BioRender.com.

Mice were intranasally (i.n.) instilled with 50 μl 12.5% DE or sterile saline 5 days/week for 3 weeks under light isoflurane sedation. The concentration of DE was previously determined by a dose-response study, which was found to elicit a strong pulmonary inflammatory response without the risk of mortality (47). Sedation for instillations was achieved using a SomnoSuite Small Animal Anesthesia System (Kent Scientific Corporation) fitted with a small animal anesthesia box. Animals were placed into the box with a flow rate of 100 mL/minute at 2.0-3.0% anesthesia. Animals were removed for instillations once breathing appeared slowed and even. Animals were held in a supine position and 50 μl of the appropriate exposure was loaded into a pipette tip and deposited at the tip of the animal’s nose one drop at a time to allow natural inhalation. Afterwards, animals were returned to their enclosures in a supine position to encourage recovery and monitored for several minutes to ensure return of normal behaviors. AT-RvD1 treated mice were administered intraperitoneal (i.p) injections of 50 μl of 250 ng AT-RvD1 or 5% ethanol (EtOH) (AT-RvD1 vehicle) in sterile saline either once per week after the 5^th^ DE instillation (weekly, for a total of 3 injections) or 5 times per week (daily, for a total of 15 injections) (**Figures 1A, B**). Dosages and controls were determined based on previously published data and a dose-response pilot study (data not shown) (32, 33, 48, 49). Animals were allowed to recover for 72 hours past-last DE instillation and AT-RvD1 injection before sacrifice.

### Animal sacrifice and sample collection

Animals were euthanized in accordance with the American Veterinary Medical Association guidelines by isoflurane overdose immediately followed by cervical dislocation, 72 hours following the final DE instillation and AT-RvD1 injection. Bronchoalveolar lavage fluid (BALF) was obtained by inserting a 25G catheter into the trachea, tying it off with suture, and lavaging the lungs three times with 1 mL of ice-cold phosphate-buffered saline (PBS) (HyClone Laboratories) for each wash. The first wash was collected in one 5 mL FACS tube while washes two and three were collected in a second 5 mL FACS tube. Both tubes were centrifuged at 300 x g for 8 minutes to pellet the cells. The supernatant fraction from wash 1 was aliquoted into a separate tube and stored at -80°C for cytokine analysis by enzyme-linked immunosorbent assay (ELISA). The supernates from washes two and three were discarded, and cell pellets from both tubes were combined with 400 µl of red blood cell lysis buffer (Life Technologies Corporation), placed on ice for 5 minutes, then centrifuged at 300 x g for 8 minutes at 4°C. The supernatant fraction was discarded, and the cell pellets were resuspended with 200 μL of PBS. A 10 μl aliquot of the cell suspension was then collected for counting using a Countess 3 FL automatic cell counter to generate total cell infiltrate values. The cell suspensions were diluted with PBS to achieve a concentration of 1x10^6^ cells/mL. 200 µl of the final cell suspension was then added to a Thermo Cytospin 4 cytocentrifuge and centrifuged at 600 rpm for 5 minutes. Slides were dried overnight for further staining and analysis. The left lobes of the lungs were tied off with suture, removed, placed in 800 μl of RNAlater (Invitrogen), and stored at -80°C until use. The right lung lobes were extracted along with the heart and tracheal tissue, then inflated with 10% neutral-buffered formalin (NBF) (Cancer Diagnostics Inc.) before hanging under 20 cm of pressure and submerged in 10% NBF overnight. They were then transferred to cassettes and stored in 10% NBF for at least 48 hours before further processing. Brains were extracted *en bloc* and immediately fixed whole in tissue cassettes in 10% NBF for at least 48 hours before further processing. All tissues were submitted to the Colorado State University Veterinary Diagnostic Laboratory Experimental Pathology Facility (EPF) for paraffin embedding and sectioning at 5 μm.

### Cellular infiltrate analysis

Staining was performed on dried slides using a Volu-Sol dip-stain kit, with methanol as the fixative, and eosin and methylene blue (Volu-Sol) as the differential stains. Slides were imaged at 20X magnification using an Olympus BX35 microscope and cellSense software version 4.1. Differential counts were performed by counting 300 cells on each image to evaluate macrophages, neutrophils, eosinophils, and lymphocytes, assisted by QuPath version 0.5.1.

### Cytokine quantification

Bronchoalveolar lavage fluid (BALF) was collected, processed, and stored as previously described above. Left lung lobes in RNAlater were homogenized with lysis buffer containing PBS, 1X RIPA lysis buffer (Thermo Scientific, Cat#: J62524.AE), and 1X proteinase inhibitor cocktail (Thermo Scientific, Cat#: 1861279) at a ratio of 50 mg tissue to 1 mL buffer. A Bead Mill 24 bead homogenizer was used to break up the tissues (speed: 5.00, time: 0:05, cycles: 3, duration: 0:03). The samples were then incubated on ice for 30 minutes, with agitation every 10 minutes. Homogenate aliquots were stored at -80°C until use. Cytokine protein concentrations in BALF and lung tissue were quantified using sandwich ELISAs. Kits were purchased from DuoSet (R&D Systems) and used according to the manufacturer’s recommendations with the modification of capture antibody diluted in BupH Carbonate-Bicarbonate Buffer (Voller’s Coating Buffer, Thermo Scientific). High-binding half-well 96-well plates (Greiner Biotech) were coated with manufacturer-specified capture antibody concentrations and incubated overnight at room temperature before progression of assay. All washes were performed using a Tecan HydroFlex™ plate washer. After completion of the assay, plates were read on a FLUOstar Omega spectrophotometer at 450 nm using Omega software version 5.7. Concentration values were calculated from standards using a four-parameter fit model in Omega MARS Software Version 4.00.

### Lung histopathology

Whole right lung coronal sections, collected as previously described above, were stained with hematoxylin and eosin (H&E) following deparaffinization and rehydration with xylene and graded EtOH solutions (xylene, 100% EtOH, 95% EtOH, 80% EtOH, 50% EtOH, and finally 1X PBS). Slides were scanned at 40X on a Vectra Polaris scanning microscope. Images of lungs from animals administered weekly AT-RvD1 injections were blinded and manually scored using a well-established semiquantitative scoring method (19, 32, 45, 46, 50). Images from animals administered daily AT-RvD1 injections were imported into VisioPharm version 2023.09.3.15043 x64 and processed through an artificial intelligence (AI) workflow for quantitative assessment of alveolar space, alveolar septa thickness, peribronchiolar inflammation, perivascular inflammation, total alveolar septa nuclei, and percentage of inducible bronchus-associated lymphoid tissue (iBALT) formations relative to tissue area. AI quantification was performed on the whole lung tissue with output measurements including area, percentages of parameters to total tissue area or total parameter counts. VisioPharm is one of the leading AI histopathology analysis software available. Our workflow and applications were designed, annotated, trained, and verified by a board-certified veterinary anatomic pathologist to identify our desired readouts and ensure accurate results. The workflow is then trained through a large set of sample images by a board-certified veterinary anatomic pathologist where structures are identified by the software and confirmed by the pathologist, allowing the software to “learn” how structures are organized and are identified in the experimental images. Briefly, the workflow consisted of multiple stepwise applications of increasing specificity, starting with identification of the lung tissue, followed by identification of airways and vessels, then inflammation and iBALT, and finally identification of airway epithelial cells and alveolar nuclei. During each step of the workflow, the results are confirmed manually to ensure accurate detection of structures. All parameters are normalized to tissue area to mitigate size and artifact skewing.

### Immunofluorescence

Whole brain sagittal paraffin embedded tissue sections, as previously described above, were deparaffinized by baking slides at 60°C for 20 minutes, followed by rehydration through an EtOH gradient (xylene, 50% xylene/50% EtOH, 100% EtOH, 95% EtOH, 70% EtOH), and then incubated in 1.0 M Tris buffered saline (TBS). Antigen retrieval was achieved using 1 nM EDTA buffer with 0.05% Tween20, pH 8.0 at 95°C for 20 minutes in a Biocare Medical Decloaking Chamber™ NxGen (Biocare Medical). Tissues were washed three times for 10 minutes each with 0.05M TBS and blocked using 2% donkey serum in 0.2% Triton-X in 1.0 M TBS for 1 hour at room temperature. Microglia were identified using rabbit anti-ionized calcium binding adaptor molecule 1 (Iba1) (1:1000; Abcam, Cat **#**: ab178846) diluted in 1.0 M TBS and incubated overnight at 4°C. Tissues were then washed four times for 10 minutes each with 0.05 M TBS. A goat anti-rabbit Alexa Fluor 647 secondary antibody (Invitrogen, Cat**#**: A21244) diluted 1:500 in 2% donkey serum in 1.0 M TBS, was applied and incubated for 1 hour at room temperature. Sections were washed three times for 10 minutes each with 0.05 M TBS. Nuclear staining was achieved by incubation with Hoechst 33342, diluted 1:2000 in PBS (Invitrogen, Cat **#**: H3570), for three minutes, then washed three times for 10 minutes each with 0.05 M M TBS. ProLong Diamond Antifade Mountant (Fisher Scientific, Cat **#**: P36970) was then applied before mounting with glass coverslips. Slides were kept at room temperature, protected from light, for 24 – 48 hours to cure mounting medium, then stored at 4°C in the dark prior to imaging. Stained sections were scanned at 40X using a Vectra Polaris microscope, and the number of microglia per area of each brain region of interest were quantified using QuPath Version 0.5.1. All sections were imaged on the same day with the same exposure settings for each channel.

### RNAscope

RNAscope^®^ assays were performed according to the formalin-fixed, paraffin-embedded (FFPE) tissue protocol provided in the RNAscope™ Multiplex Fluorescent Reagent Kit v2 Assay Manual (Document Number: UM 323100). Mouse lung and brain sections previously described above were utilized, with 3 animals per sex, per treatment condition, per genotype selected. Probes for IL-1β (Cat #: 316891-C3), CXCL10 (Cat #: 408921-C3), IL-10 (Cat #: 317261-C2), TGF-β (Cat #: 407751), and AREG (Cat #: 430501) were purchased from Advanced Cell Diagnostics, Inc. TSA Vivid Fluorophore dyes 570 (Cat #: 323272) and 650 (Cat #: 232273) were purchased from Advanced Cell Diagnostics, Inc. and diluted in TSA buffer 1:1500 (Cat #: 322809). Opal Polaris 780 Fluorophore Reagent Pack was purchased from Akoya Biosciences (Cat #: FP1501001KT). TSA-DIG was diluted 1:1500 in TSA buffer for lung sections and 1:750 for brain sections. Opal Polaris 780 Fluorophore was diluted 1:500 in Antibody Diluent/Block (Akoya Biosciences, Cat #: ARD1001EA) for lung sections and 1:200 for brain sections. Positive control slides using RNAscope™ 3-plex positive control probes (CAT #: 320881) and negative control slides using RNAscope™ 3-plex negative control probes (Cat#: 320871) were utilized to validate the assay and results. After completion of the RNAscope^®^ protocol, sections were incubated with RNAscope^®^ DAPI (Advanced Cell Diagnostics, Inc.) for 30 seconds, then mounted on glass coverslips with ProLong Diamond Antifade mounting medium (Invitrogen). The mounted sections were kept at room temperature, protected from light, for 24–48 hours to cure mounting medium, before being stored at 4°C until imaging. Slides were scanned at 40X using a Vectra Polaris microscope, with all sections imaged on the same day using the same exposure settings for each channel. QuPath Version 0.5.1 was used according to manufacturer protocols to detect the cell nuclei and quantify subcellular dots per nucleus for the entirety of the lung tissue and for each of the brain regions of interest.

### Statistical analysis

All statistical and graphical analyses were performed using GraphPad Prism Version 10. Outliers were tested for and removed from the datasets using ROUT analysis with Q=1%. Statistical significance was determined by performing 3-way ANOVA analyses with Benjamini, Krieger and Yekutieli *post-hoc* analysis for pairwise comparisons to reduce false discovery rate. A p-value of less than or equal to 0.05 was used to determine significance and a p-value of less than or equal to 0.1 was considered a trend. Individual animals are represented by a single symbol on figures, with filled shapes representing male animals and unfilled shapes representing female animals. On figures, significance is denoted by, * = *p* ≤ 0.05; ** = *p* ≤ 0.01; *** = *p* ≤ 0.001; **** = *p* ≤ 0.0001.

## Results

### IL-22 KO alters lung immune cell trafficking and weekly AT-RvD1 administration reduces cellular infiltration in IL-22 KO mice following repetitive organic dust exposure

**Figure 2:**
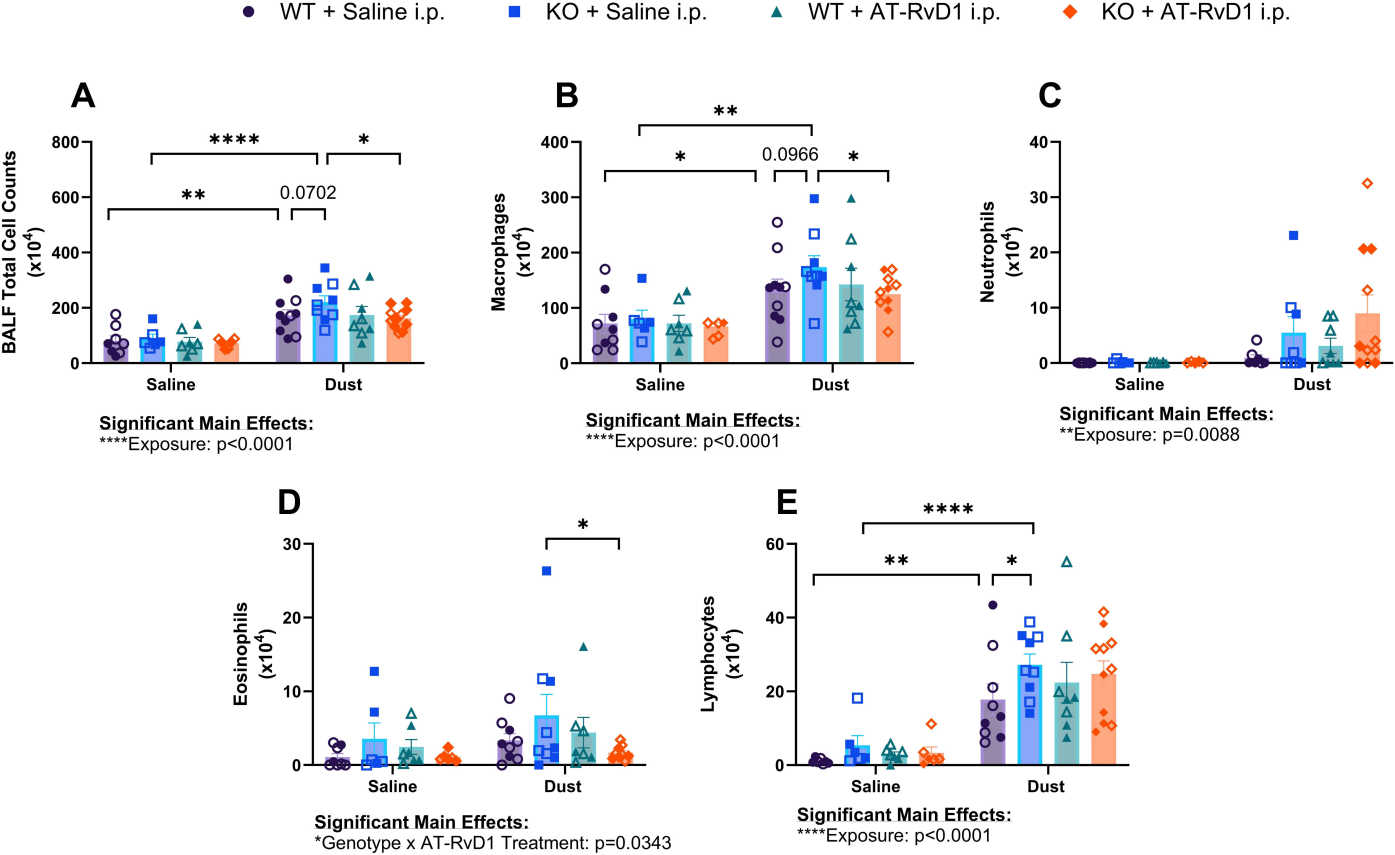
Impacts of DE and AT-RvD1 on BALF immune cellular infiltrates in WT and IL-22 KO animals. WT and KO animals were instilled i.n. with 12.5% DE 5 days/week for 3 weeks and treated with 250 ng AT-RvD1 i.p. once/week for 3 weeks. **(A)** total cell counts, **(B)** macrophages, **(C)** neutrophils, **(D)** eosinophils, **(E)** lymphocytes. 3 way ANOVA with Benjamini, Krieger and Yekutieli *post-hoc* analysis, error bars = SEM; * = p ≤ 0.05; ** = p ≤ 0.01; **** = p ≤ 0.0001. Sample sizes: WT Saline i.n. + Saline i.p (3 female/3 male), WT Saline i.n. + AT-RvD1 i.p (3 female/3 male), WT Dust i.n. + Saline i.p (5 female/5 male), WT Dust i.n. + AT-RvD1 i.p (4 female/4 male), KO Saline i.n. + Saline i.p (3 female/3 male), KO Saline i.n. + AT-RvD1 i.p (3 female/3 male), KO Dust i.n. + Saline i.p (5 female/4 male), KO Dust i.n. + AT-RvD1 i.p (6 female/5 male).

**Figure 3:**
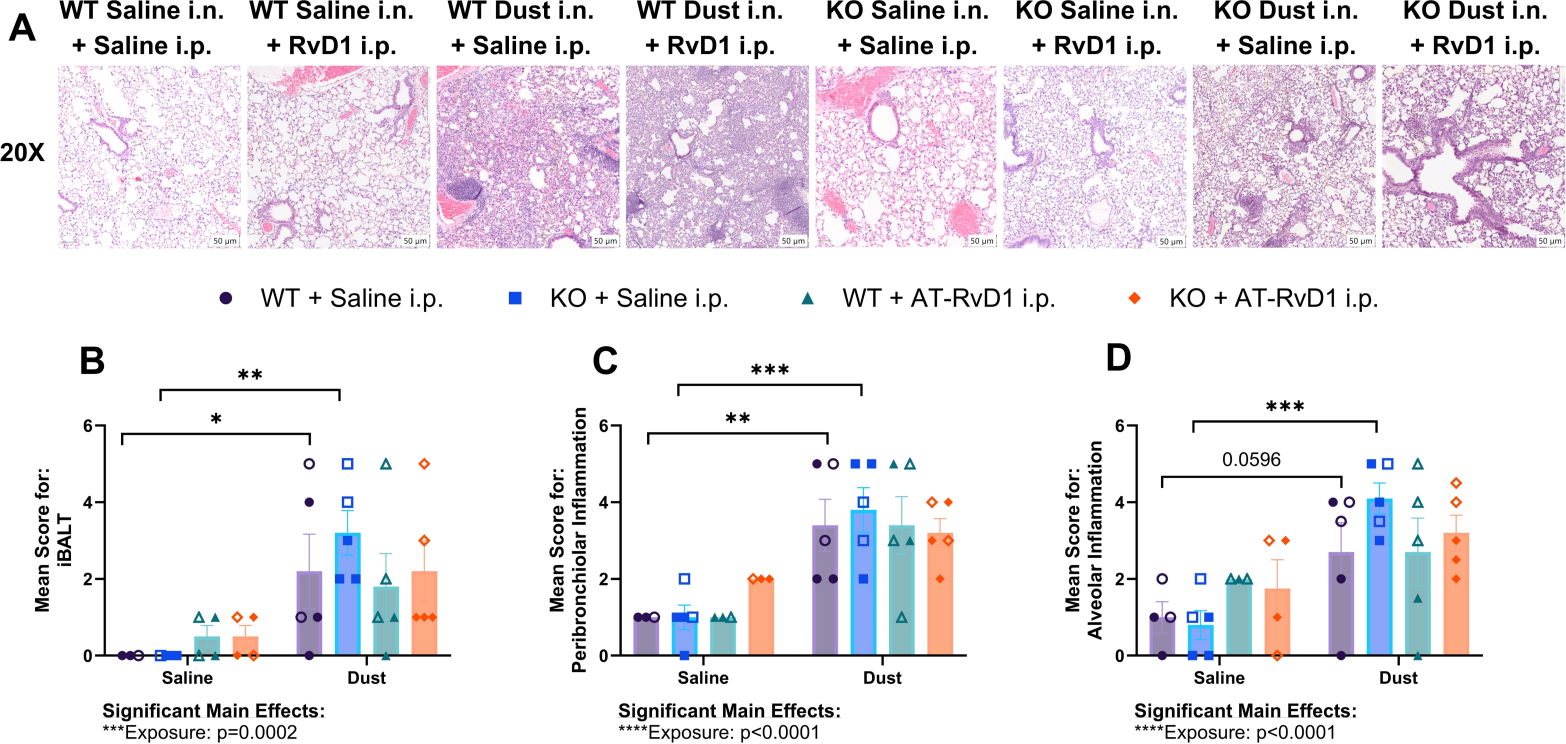
AT-RvD1 administration once weekly does not improve lung pathology. WT and KO animals were instilled i.n. with 12.5% DE 5 days/week for 3 weeks and treated with 250 ng AT-RvD1 i.p. once/week for 3 weeks. **(A)** representative images at 20X magnification, **(B)** mean inflammatory score for iBALT, **(C)** mean inflammatory score for peribronchiolar inflammation, **(D)** mean inflammatory score for alveolar inflammation. Scale bar = 50 μm. 3 way ANOVA with Benjamini, Krieger and Yekutieli *post-hoc* analysis, error bars = SEM; * = p ≤ 0.05; ** = p ≤ 0.01; *** = p ≤ 0.001. Sample sizes: WT Saline i.n. + Saline i.p (2 female/2 male), WT Saline i.n. + AT-RvD1 i.p (2 female/2 male), WT Dust i.n. + Saline i.p (2 female/3 male), WT Dust i.n. + AT-RvD1 i.p (3 female/3 male), KO Saline i.n. + Saline i.p (3 female/3 male), KO Saline i.n. + AT-RvD1 i.p (2 female/2 male), KO Dust i.n. + Saline i.p (3 female/3 male), KO Dust i.n. + AT-RvD1 i.p (3 female/3 male).

We have previously demonstrated that DE exposure leads to increased infiltration of immune cells into the airways (19, 32, 51). One well-established mechanism of AT-RvD1 is its ability to modify immune cell recruitment to sites of infection and injury (49). We aimed to establish an effective dosing strategy to assess the therapeutic applications of AT-RvD1 in a model of repetitive ODE. WT and IL-22 KO mice were instilled i.n. with 50 μl of either 12.5% DE in sterile saline or sterile saline for 5 days per week for 3 weeks (15 total installations) and injected i.p. with 50 μl of either 250 ng AT-RvD1 in sterile saline or 5% EtOH (AT-RvD1 carrier) in sterile saline once per week following the 5^th^ DE instillation each week (3 total injections) (**Figure 1A**). To determine the impact of AT-RvD1 treatment on immune cell recruitment following DE, we examined cellular infiltration into the airway via BALF cellular analysis (**Figures 2A–E**) and lung histopathology (**Figures 3A–D**).

Upon analysis of BALF cellular infiltrates, we observed an increase in the total number of cellular infiltrates in both WT (p=0.0011) and KO (p<0.0001) mice exposed to DE compared to saline controls (**Figure 2A**). Additionally, we observed a trend of increased total cells in KO mice exposed to DE compared to WT mice exposed to DE (p=0.0702) (**Figure 2A**). KO mice exposed to DE and treated with AT-RvD1 exhibited reduced total cell counts compared to KO mice exposed to DE and treated with saline (p=0.0265) (**Figure 2A**). Macrophage differential counts revealed increased influx in WT (p=0.0162) and KO (p=0.0015) mice exposed to DE compared to WT and KO mice exposed to saline, respectively (**Figure 2B**). We also observed a trend between KO mice exposed to DE and WT mice exposed to DE (p=0.0966) (**Figure 2B**). KO mice exposed to DE and treated with AT-RvD1 demonstrated significantly reduced macrophage differential counts compared to KO mice exposed to DE and treated with AT-RvD1 (p=0.0457) (**Figure 2B**). We did not observe any significant differences or trends in the number of neutrophils, regardless of exposure or treatment condition (**Figure 2C**). Eosinophil differential counts revealed a decrease in the number of eosinophil influx in KO mice exposed to DE and treated with AT-RvD1 compared to KO mice exposed to DE and treated with saline (p=0.0180) (**Figure 2D**). We also observed elevated trafficking of lymphocytes in WT (p=0.0013) and KO (p<0.0001) mice exposed to DE compared to saline exposed controls as well as significantly increased lymphocyte counts in KO mice exposed to DE compared to WT mice exposed to DE (p=0.0454) (**Figure 2E**).

We also examined lung pathology of mice injected with 250 ng AT-RvD1 once per week to assess the effectiveness of AT-RvD1 at reducing DE-induced lung pathology. We evaluated inducible bronchus associated lymphoid tissue (iBALT), peribronchiolar inflammation, and alveolar inflammation using a semiquantitative scoring method previously utilized in our laboratory (19, 32, 50, 52, 53). Statistical analysis revealed a significant main effect of DE exposure in all three parameters evaluated: iBALT (p=0.0002), peribronchiolar inflammation (p<0.0001) and alveolar inflammation (p<0.0001) (**Figures 3B–D**). However, on *post-hoc* analysis, we observed no statistically significant differences or trends between AT-RvD1 i.p. and saline i.p. treatment groups (**Figures 3B–D**). Interestingly, we observed a similar pattern in all parameters evaluated. We found a significant increase in the mean inflammatory score of iBALT in both WT (p=0.0446) and KO (p=0.0025) animals exposed to DE compared to saline-exposure controls (**Figure 3B**). Additionally, we observed a significantly increased peribronchiolar inflammation in WT (p=0.0064) and KO (p=0.0005) animals exposed to DE compared to saline-exposed controls (**Figure 3C**), as well as a significant increase in the mean score for alveolar inflammation in KO animals exposed to DE compared to saline-exposure controls (p=0.0004) and a trend in WT animals exposed to DE compared to saline-exposure controls (p=0.0596) (**Figure 3D**). We also did not observe any significant changes in mean pathological scores of WT compared to KO animals regardless of exposure or treatment group.

Following our observations of no statistically significant changes in animals exposed to DE and treated with AT-RvD1 once weekly (**Figures 3A–D**), we developed a 5 day/week AT-RvD1 injection regimen, which we found yielded more significant therapeutic efficacy, through histopathology resolution, and was thus employed as our model for further analysis of the pulmonary and neurological inflammatory response to DE. Briefly, WT and IL-22 KO mice were instilled i.n. with 50 μl of either 12.5% DE in sterile saline or sterile saline for 5 days per week for 3 weeks, and then injected i.p. with 50 μl of either 250 ng AT-RvD1 in sterile saline or 5% EtOH (AT-RvD1 carrier) in sterile saline 5 days per week for 3 weeks (**Figure 1B**).

**Figure 4:**
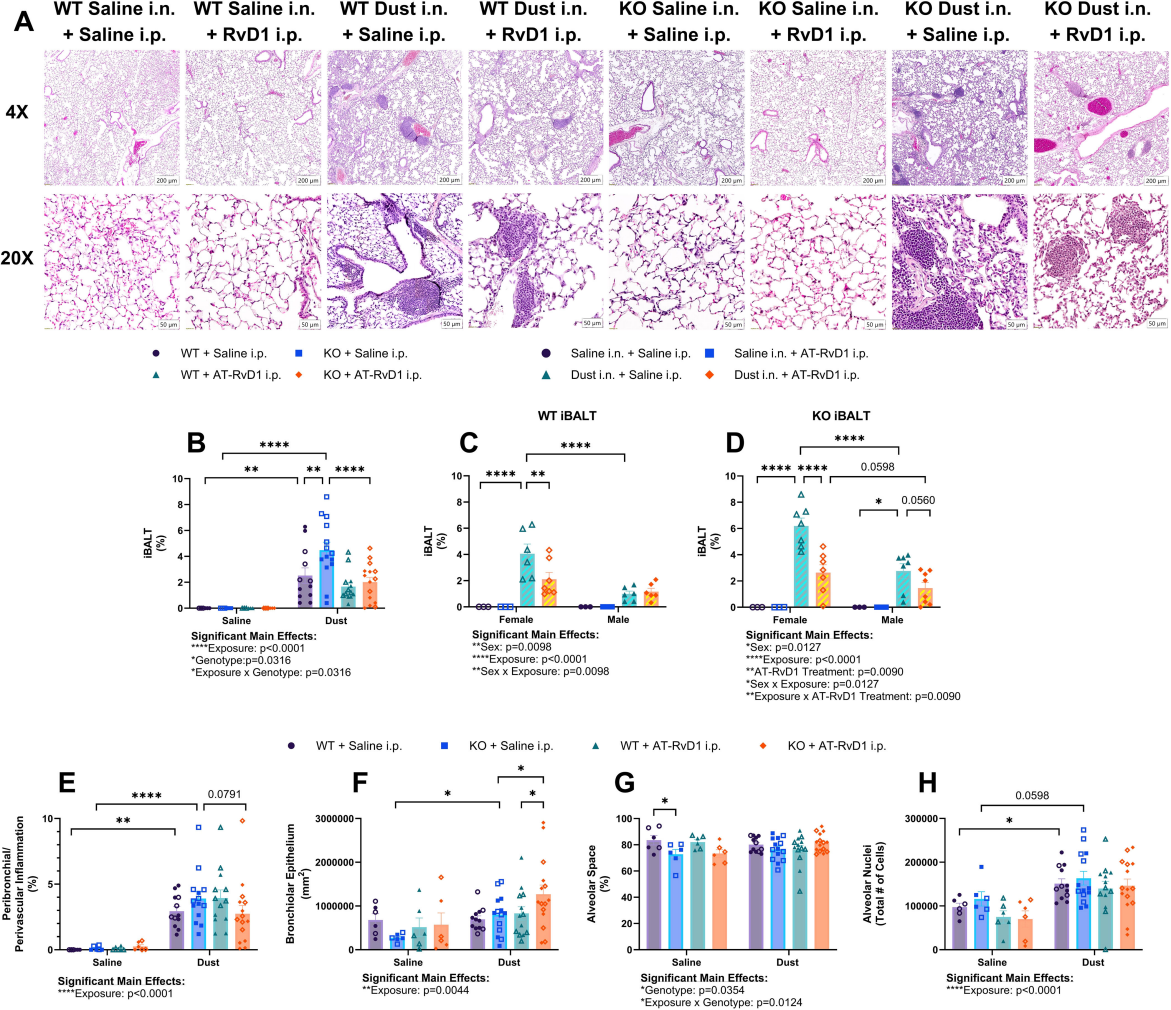
AT-RvD1 treatment daily reduces iBALT in animals exposed to DE. WT and KO animals were instilled i.n. with 12.5% DE 5 days/week for 3 weeks and treated with 250 ng AT-RvD1 i.p. 5 days/week for 3 weeks. **(A)** representative images at 4X and 10X magnification, **(B)** iBALT percentage in all animals, **(C)** iBALT percentage in WT animals by sex, **(D)** iBALT percentage in KO animals by sex, **(E)** combined peribronchiolar and perivascular inflammation percentage in all animals, **(F)** bronchiolar epithelium area, **(G)** alveolar space percentage, **(H)** total alveolar nuceli. Scale bar = 50 μm. 3 way ANOVA with Benjamini, Krieger and Yekutieli *post-hoc* analysis, error bars = SEM; * = p ≤ 0.05; ** = p ≤ 0.01; **** = p ≤ 0.0001. Sample sizes: WT Saline i.n. + Saline i.p (3 female/3 male), WT Saline i.n. + AT-RvD1 i.p (3 female/3 male), WT Dust i.n. + Saline i.p (6 female/6 male), WT Dust i.n. + AT-RvD1 i.p (6 female/6 male), KO Saline i.n. + Saline i.p (3 female/3 male), KO Saline i.n. + AT-RvD1 i.p (3 female/3 male), KO Dust i.n. + Saline i.p (7 female/7 male), KO Dust i.n. + AT-RvD1 i.p (7 female/8 male).

Our analysis of lung histopathology via VisioPharm AI applications revealed an increase in the percentage of iBALT relative to lung tissue area in both WT (p=0.0014) and KO mice (p<0.0001) exposed to DE (**Figure 4B**). Interestingly, we also observed a significant increase in iBALT percentage in KO mice compared to WT mice exposed to DE (p=0.0002) (**Figure 4B**). Additionally, KO mice exposed to DE and treated with AT-RvD1 displayed a significant decrease in iBALT percent of lung tissue compared to KO mice exposed to DE and treated with saline (p<0.0001) (**Figure 4B**). Furthermore, we discovered significant sex differences in the iBALT percentages displayed by both WT and KO animals. We found that female animals exposed to DE and treated with saline exhibited a significantly higher iBALT percentage than male animals exposed to DE and treated with saline in both WT (p<0.0001) and KO (p<0.0001) animals (**Figures 4C, D**). Additionally, both WT (p=0.0021) and KO (p<0.0001) female animals exposed to dust and treated with AT-RvD1 displayed significantly reduced iBALT compared to saline treated female animals (**Figures 4C, D**). Evaluation of peribronchiolar and perivascular inflammation percentage revealed a significant increase in both WT (p=0.0013) and KO (p<0.0001) mice exposed to DE (**Figure 4E**). We observed a trend of reduced peribronchiolar and perivascular inflammation in KO animals exposed to DE and treated with saline and KO mice exposed to DE and treated with AT-RvD1 (p=0.0791) (**Figure 4E**). Bronchiolar epithelium area in KO mice exposed to DE was significantly increased compared to KO mice exposed to saline (p=0.0474) and was further increased in KO mice exposed to DE and treated with AT-RvD1 compared to KO mice exposed to DE and treated with saline (p=0.0233) (**Figure 4F**). Bronchiolar epithelium area was also decreased in WT mice exposed to DE and treated with AT-RvD1 compared to KO mice exposed to DE and treated with AT-RvD1 (p=0.0353) (**Figure 4F**). We observed a significant decrease in alveolar air space in KO mice exposed to saline compared to WT mice exposed to saline (p=0.0301) (**Figure 4G**). Total alveolar nuclear counts were observed to be elevated in WT mice exposed to DE compared to WT mice exposed to saline (p=0.0377), with an observed trend of increased nuclei in KO mice exposed to DE compared to KO mice exposed to saline (p=0.0598) (**Figure 4H**).

### Impacts of AT-RvD1 on lung inflammatory mediator production in WT and IL-22 KO mice during recovery following repetitive DE exposure

**Figure 5:**
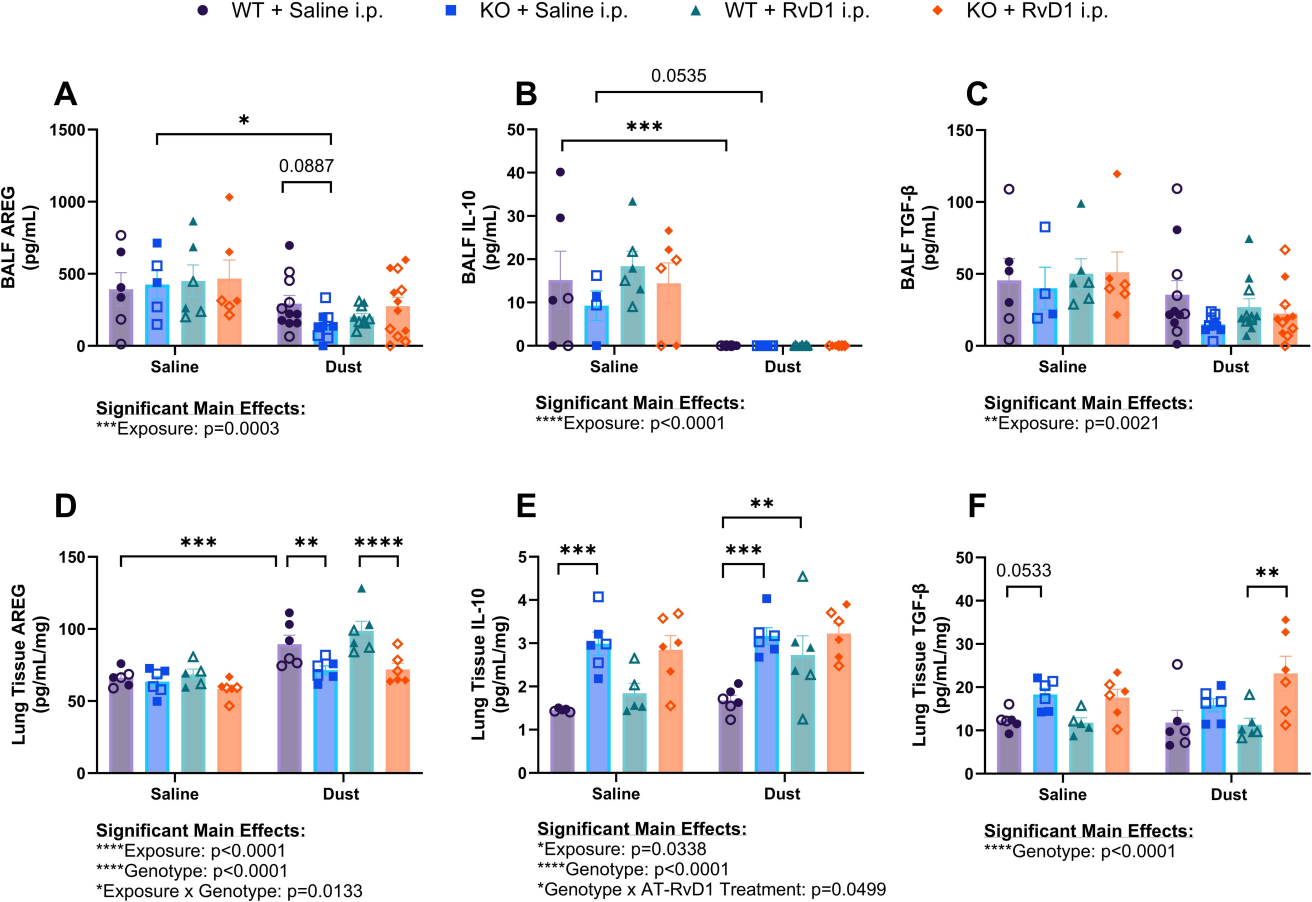
Effects of repetitive dust exposure and RvD1 treatment on bronchoalveolar lavage fluid (BALF) and lung tissue homogenate cytokines. WT and KO animals were instilled i.n. with 12.5% DE 5 days/week for 3 weeks and treated with 250 ng AT-RvD1 i.p. 5 days/week for 3 weeks. **(A)** amphiregulin (AREG) concentrations in BALF, **(B)** interleukin-10 (IL-10) concentrations in BALF, **(C)** transforming growth factor-β (TGF-β) concentrations in BALF, **(D)** AREG concentrations in lung tissue, **(E)** IL-10 concentrations in lung tissue, and **(F)** TGFβ concentrations in lung tissue. 3 way ANOVA with Benjamini, Krieger and Yekutieli *post-hoc* analysis, error bars = SEM; * = p ≤ 0.05; ** = p ≤ 0.01; *** = p ≤ 0.001; **** = p ≤ 0.0001. BALF sample sizes: WT Saline i.n. + Saline i.p (3 female/3 male), WT Saline i.n. + AT-RvD1 i.p (3 female/3 male), WT Dust i.n. + Saline i.p (6 female/6 male), WT Dust i.n. + AT-RvD1 i.p (6 female/6 male), KO Saline i.n. + Saline i.p (3 female/3 male), KO Saline i.n. + AT-RvD1 i.p (3 female/3 male), KO Dust i.n. + Saline i.p (7 female/7 male), KO Dust i.n. + AT-RvD1 i.p (7 female/8 male). Lung tissue sample sizes: WT Saline i.n. + Saline i.p (3 female/3 male), WT Saline i.n. + AT-RvD1 i.p (3 female/3 male), WT Dust i.n. + Saline i.p (3 female/3 male), WT Dust i.n. + AT-RvD1 i.p (3 female/3 male), KO Saline i.n. + Saline i.p (3 female/3 male), KO Saline i.n. + AT-RvD1 i.p (3 female/3 male), KO Dust i.n. + Saline i.p (3 female/3 male), KO Dust i.n. + AT-RvD1 i.p (3 female/3 male).

We have previously demonstrated that ODE in mice increases both pro- and anti-inflammatory cytokine production (32, 45, 47). In our recovery model, we found that after 15 DE instillations, mice displayed altered cytokine and inflammatory mediator production in the airway and pulmonary tissue compartments at the protein level (**Figures 5A–F**).

Evaluation of amphiregulin (AREG) concentrations in BALF showed that KO mice exposed to DE exhibited decreased concentrations compared to saline controls (p=0.0130) and that KO mice exposed to DE displayed a trend of decreased AREG concentrations compared to WT mice exposed to DE (p=0.0887) (**Figure 5A**). Concentrations of interleukin-10 (IL-10), a classic anti-inflammatory, pro-resolution cytokine was decreased in the BALF of WT mice exposed to DE compared to saline controls (p=0.0009) and a trend of decreased concentrations in KO mice exposed to DE compared to saline controls (p=0.0535) was also observed (**Figure 5B**). Transforming growth factor-β (TGF-β) concentrations in BALF did not yield any significant differences regardless of genotype or treatment groups, however we did observe a significant main effect of DE exposure (p=0.0021) (**Figure 5C**). In the tissue compartment, IL-10 was observed to be elevated in KO mice exposed to saline compared to WT mice exposed to saline (p=0.0003) and in KO mice exposed to DE compared to WT mice exposed to DE (p=0.0002). WT mice exposed to DE and treated with AT-RvD1 exhibited increased tissue IL-10 concentrations compared to WT mice exposed to DE and treated with saline (p=0.0070) (**Figure 5D**). AREG quantification in lung tissues of WT mice exposed to DE displayed increased concentrations compared to WT saline controls (p=0.0005) and were observed to be decreased in KO mice exposed to DE compared to WT mice exposed to DE (p=0.0051) (**Figure 5E**). We also observed a significant decrease in AREG concentrations in KO mice exposed to DE and treated with AT-RvD1 compared to WT mice exposed to DE and treated with AT-RvD1 (p<0.0001). Lung tissue TGF-β concentrations demonstrated a trend of increased concentrations in KO mice exposed to saline compared to WT mice exposed to saline (p=0.0533) and a significant increase in KO mice exposed to DE and treated with AT-RvD1 compared to WT mice exposed to DE and treated with AT-RvD1 (p<0.0003) (**Figure 5F**).

**Figure 6:**
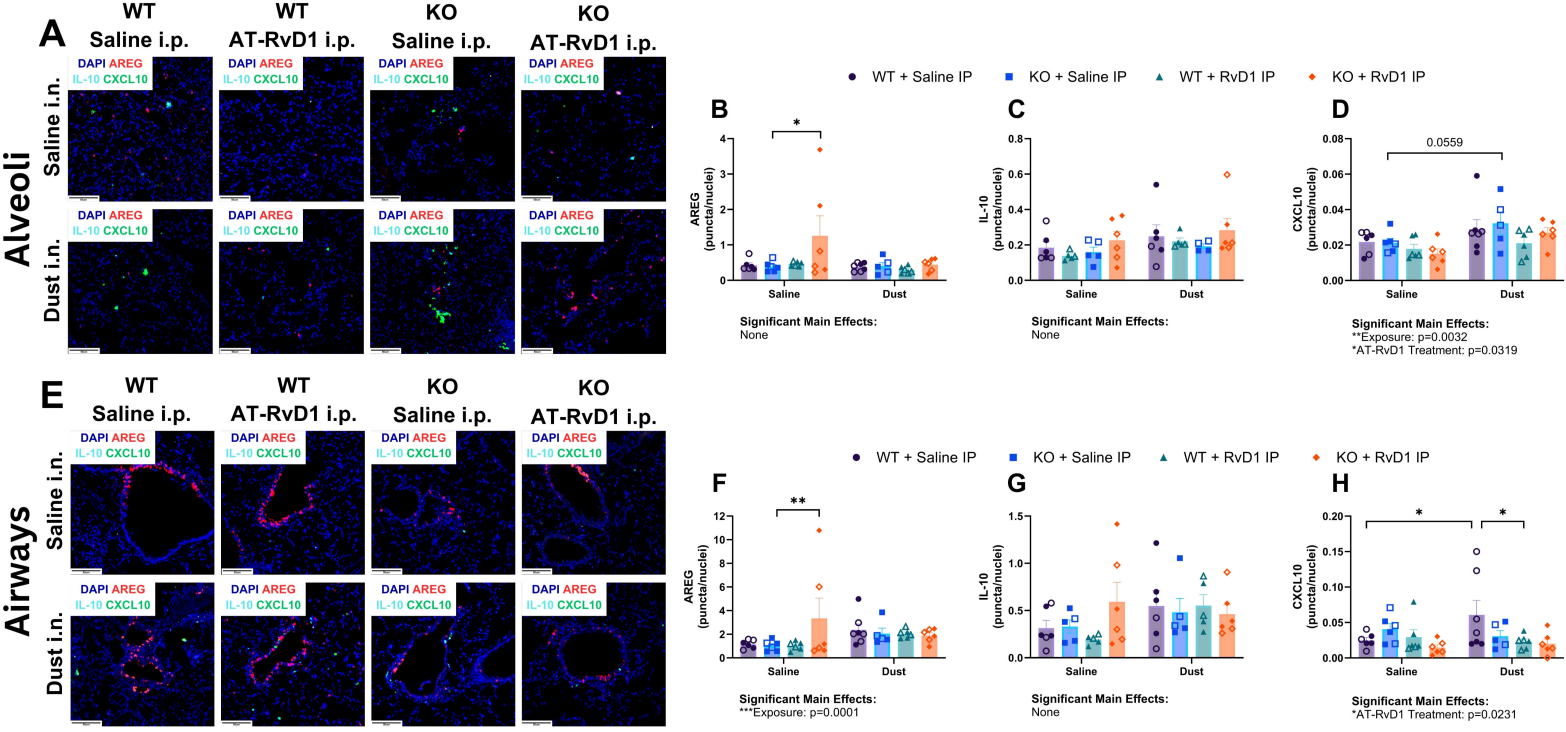
AT-RvD1 treatment reduces cxcl10 mRNA expression in WT animals exposed to DE. WT and KO animals were instilled i.n. with 12.5% DE 5 days/week for 3 weeks and treated with 250 ng AT-RvD1 i.p. 5 days/week for 3 weeks. **(A)** representative images of the alveolar compartment at 40X magnification, **(B)** areg expression, **(C)** il10 expression, **(D)** cxcl10 expression, **(E)** representative images of the airway compartment at 40X magnification, **(F)** areg expression, **(G)** il10 expression, **(H)** cxcl10 expression. Scale bar = 50 μm. 3 way ANOVA with Benjamini, Krieger and Yekutieli *post-hoc* analysis, error bars = SEM; * = p ≤ 0.05; ** = p ≤ 0.01. Sample sizes: WT Saline i.n. + Saline i.p (3 female/3 male), WT Saline i.n. + AT-RvD1 i.p (3 female/3 male), WT Dust i.n. + Saline i.p (4 female/3 male), WT Dust i.n. + AT-RvD1 i.p (3 female/3 male), KO Saline i.n. + Saline i.p (3 female/3 male), KO Saline i.n. + AT-RvD1 i.p (3 female/3 male), KO Dust i.n. + Saline i.p (3 female/3 male), KO Dust i.n. + AT-RvD1 i.p (3 female/3 male).

To evaluate mRNA transcript expression in animals exposed to DE and treated with AT-RvD1, we utilized RNAscope technology to visualize and quantify mRNA transcripts in whole lung tissue sections. We evaluated *areg*, *il10*, and C-X-C motif chemokine ligand 10 (*cxcl10*) transcripts in the alveolar and airway compartments to assess the recovery of IL-22 KO mice and the therapeutic impacts of AT-RvD1 and to assess the differences between alveolar and airway inflammatory markers (**Figures 6A–E**). In the alveolar compartment, we observed a significant increase in *areg* expression in KO mice exposed to saline and treated with AT-RvD1 compared to KO mice exposed to saline and treated with saline (p=0.0051) (**Figure 6B**). Assessment of *il10* expression in the alveolar compartment yielded no significant differences or trends of, regardless of DE exposure or AT-RvD1 treatment or genotype (**Figure 6C**). Expression of *cxcl10* in the alveolar compartments of KO mice exposed to DE displayed a trend of increased expression compared to saline-exposed KO controls (p=0.0559) (**Figure 6D**). In the airway compartment, we again observed a significant increase in *areg* expression in KO mice exposed to saline and treated with AT-RvD1 compared to KO mice exposed to saline and treated with saline (p=0.0187) (**Figure 6F**). We again observed no significant differences or trends of *il10* expression in the airway compartment (**Figure 6G**). Interestingly, we did observe significantly elevated *cxcl10* expression in WT mice exposed to DE compared to saline-exposed WT controls (p=0.0156), which was then revealed to be decreased in WT animals exposed to DE and treated with AT-RvD1 (p=0.0107) (**Figure 6H**).

### Agriculture dust exposure is associated with gliosis and AT-RvD1 administration reduces neuroinflammation

**Figure 7:**
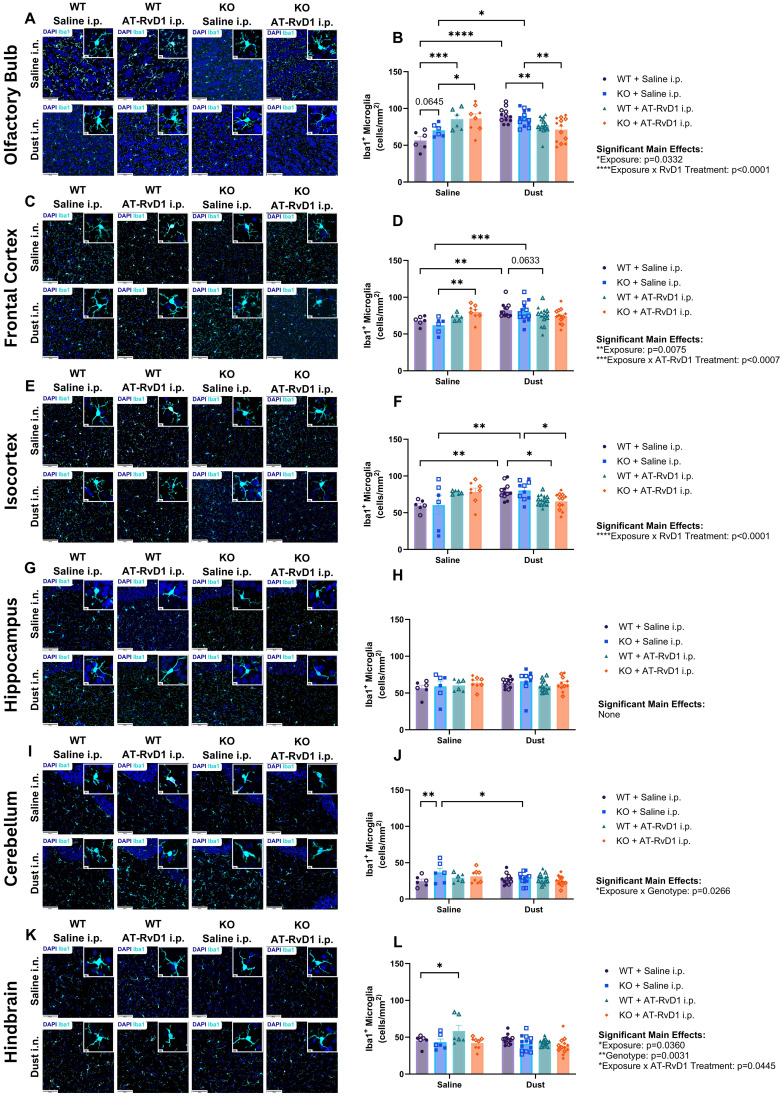
Gliosis is associated with agriculture dust exposure. WT and KO animals were instilled i.n. with 12.5% DE 5 days/week for 3 weeks and treated with 250 ng AT-RvD1 i.p. 5 days/week for 3 weeks. **(A)** representative images of the olfactory bulb at 40X magnification, **(B)** microglia counts in the olfactory bulb, **(C)** representative images of the frontal cortex at 40X magnification, **(D)** microglia counts in the frontal cortex, **(E)** representative images of the isocortex at 40X magnification, **(F)** microglia counts in the isocortex, **(G)** representative images of the hippocampus at 40X magnification, **(H)** microglia counts in the hippocampus, **(I)** representative images of the cerebellum at 40X magnification, **(J)** microglia counts in the cerebellum, **(K)** representative images of the hindbrain at 40X magnification, **(L)** microglia counts in the hindbrain. Scale bar = 50 μm and 5 μm. 3 way ANOVA with Benjamini, Krieger and Yekutieli *post-hoc* analysis, error bars = SEM; * = p ≤ 0.05; ** = p ≤ 0.01; *** = p ≤ 0.001; **** = p ≤ 0.0001. WT Saline i.n. + Saline i.p (3 female/3 male), WT Saline i.n. + AT-RvD1 i.p (3 female/3 male), WT Dust i.n. + Saline i.p (6 female/6 male), WT Dust i.n. + AT-RvD1 i.p (8 female/6 male), KO Saline i.n. + Saline i.p (3 female/3 male), KO Saline i.n. + AT-RvD1 i.p (4 female/4 male), KO Dust i.n. + Saline i.p (7 female/6 male), KO Dust i.n. + AT-RvD1 i.p (6 female/7 male).

Brains of WT and IL-22 KO animals exposed to DE and treated with AT-RvD1 were assessed for microglia proliferation via immunofluorescence for Iba1^+^ cells. Cells were counted and represented as a function of the area of each brain region of interest: olfactory bulb, frontal cortex, isocortex, hippocampus, cerebellum, and hindbrain. The olfactory bulb was chosen due to its close proximity to the nasal passages and based upon previous investigations that have shown that intranasal instillations of other particulate matter sources result in gliosis in this region (54). The frontal cortex, isocortex, and hippocampus regions of interest were evaluated due to their functions involving memory and cognition, the decline of which is a hallmark of neurodegenerative disease. The cerebellum was chosen as it controls motor function, and changes in motor function can also be a pathological marker seen in neurodegenerative disease. The hindbrain region of the brain stem is the innervation site for the vagus nerve, which has been implicated in neuroinflammatory models of chronic lung inflammation (2). Increased numbers of Iba1^+^ microglia were detected in areas involved in olfactory sensing and cognition including the olfactory bulb, frontal cortex, and isocortex in WT and KO mice (**Figures 7A–F**). KO mice displayed increased Iba1^+^ microglia in the cerebellum, which is associated with motor function (**Figures 7I, J**). These data indicate that KO mice have altered responses to DE compared to WT controls, but that all mice regardless of genotype, experienced microgliosis in their brains following DE exposure. The increased number of microglia cells detected in brain tissue, is a hallmark sign of neuroinflammation and is a reliable marker for detecting neuroinflammatory processes (55–57).

Evaluation of the olfactory bulbs of animals exposed to DE revealed significant increases in the number of microglia in both WT (p<0.0001) and KO (p=0.0109) animals. We also observed an increase in the number of microglia in mice exposed to saline and treated with AT-RvD1 compared to with saline-exposed, saline-treated controls in both WT (p=0.0002) and KO (p=0.0223) genotypes (**Figure 7B**). Additionally, in the olfactory bulbs we observed a trend of increased microglia numbers in KO mice exposed to saline and treated with saline compared to their WT saline-exposed, saline-treated controls (0.0645) (**Figure 7B**). Animals exposed to DE and treated with AT-RvD1 displayed decreased numbers of Iba1+ cells compared to DE-exposed, saline-treated controls in both WT (p=0.0090) and KO (p=0.0027) genotypes (**Figure 7B**). In the frontal cortex, both WT (p=0.0094) and KO (p=0.0009) animals exposed to DE displayed increased microglia numbers (**Figure 7D**). Saline-exposed, AT-RvD1-treated KO animals also displayed increased numbers compared to saline-exposed, saline-treated KO animals (p=0.0030) (**Figure 7D**). DE-exposed, AT-RvD1-treated WT animals displayed a trend of decreased numbers compared to WT DE-exposed, saline-treated animals (p=0.0633) (**Figure 7D**). Microglia numbers in the isocortex revealed a similar pattern as the olfactory bulb, with both WT (p=0.0051) and KO (p=0.0052) animals exposed to DE and treated with saline demonstrating increased microglia numbers compared to their saline controls (**Figure 7F**). We did not observe any significant differences in the number of microglia in the hippocampus, regardless of exposure or treatment condition (**Figures 7G, H**). Additionally, animals treated with AT-RvD1 exhibited decreased microglia numbers compared to their saline-treated controls for both WT (p=0.0247) and KO (p=0.0117) animals (**Figure 7F**). Saline-exposed KO animals displayed increased microglia counts in their cerebellums compared to WT saline-exposed animals (p=0.0093) and increased counts in DE-exposed, saline treated KO animals compared to saline-exposed controls (p=0.0313) (**Figures 7I, J**). WT animals exposed to saline and treated with AT-RvD1 displayed increased microglia numbers compared to saline-exposed, saline-treated WT animals in the hindbrain (p=0.0253) (**Figures 7K, L**).

### AT-RvD1 treatment alters brain transcript expression in animals exposed to DE

**Figure 8:**
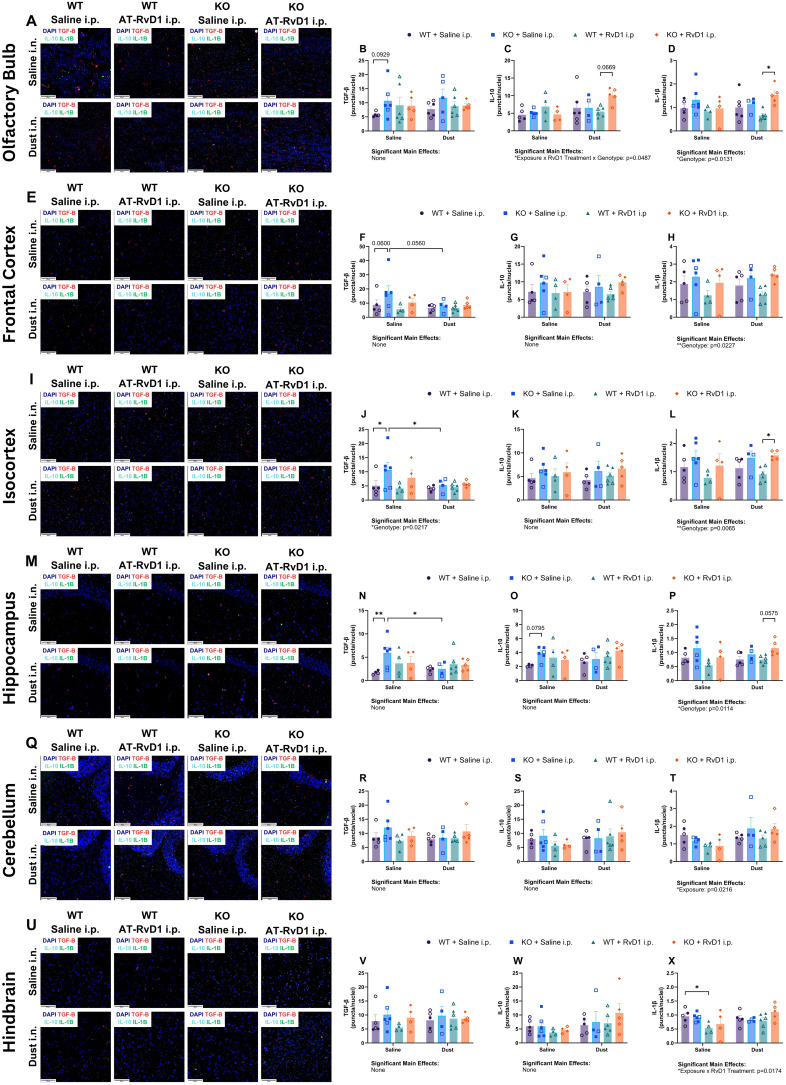
IL-22 knock-out alters brain mRNA transcripts in the presence of AT-RvD1 and DE. WT and KO animals were instilled i.n. with 12.5% DE 5 days/week for 3 weeks and treated with 250 ng AT-RvD1 i.p. 5 days/week for 3 weeks. **(A)** representative images of the olfactory bulb at 40X magnification, **(B)** tgfb expression in the olfactory bulb, **(C)** il10 expression in the olfactory bulb, **(D)** il1b expression in the olfactory bulb, **(E)** representative images of the frontal cortex at 40X magnification, **(F)** tgfb expression in the frontal cortex, **(G)** il10 expression in the frontal cortex, **(H)** il1b expression in the frontal cortex, **(I)** representative images of the isocortex at 40X magnification, **(J)** tgfb expression in the isocortex, **(K)** il10 expression in the isocortex, **(L)** il1b expression in the isocortex, **(M)** representative images of the hippocampus at 40X magnification, **(N)** tgfb expression in the hippocampus, **(O)** il10 expression in the hippocampus, **(P)** il1b expression in the hippocampus, **(Q)** representative images of the cerebellum at 40X magnification, **(R)** tgfb expression in the cerebellum, **(S)** il10 expression in the cerebellum, **(T)** il1b expression in the cerebellum, **(U)** representative images of the hindbrain at 40X magnification, **(V)** tgfb expression in the hindbrain, **(W)** il10 expression in the hindbrain, **(X)** il1b expression in the hindbrain. Scale bar = 50 μm. 3 way ANOVA with Benjamini, Krieger and Yekutieli *post-hoc* analysis, error bars = SEM; * = p ≤ 0.05; ** = p ≤ 0.01. Sample sizes: WT Saline i.n. + Saline i.p (3 female/3 male), WT Saline i.n. + AT-RvD1 i.p (2 female/2 male), WT Dust i.n. + Saline i.p (3 female/3 male), WT Dust i.n. + AT-RvD1 i.p (3 female/3 male), KO Saline i.n. + Saline i.p (3 female/3 male), KO Saline i.n. + AT-RvD1 i.p (2 female/2 male), KO Dust i.n. + Saline i.p (2 female/2 male), KO Dust i.n. + AT-RvD1 i.p. (3 female/3 male).

We again utilized RNAscope technology to visualize and quantify mRNA transcript expression in WT and KO animals exposed to DE and treated with AT-RvD1 using whole brain tissue sections to evaluate individual regions of interest. We evaluated *tgfb*, *il10*, and *il1β* transcripts in the olfactory bulb, frontal cortex, isocortex, hippocampus, cerebellum, and hindbrain (**Figures 8A–X**).

Transcript quantification in the olfactory bulb revealed a trend of increased *tgfb* expression between WT saline-exposed mice and KO saline-exposed mice (p=0.0929) (**Figure 8B**). Additionally, a trend was observed for increased *il10* transcription in KO mice exposed to DE and treated with AT-RvD1 compared to WT mice exposed to DE and treated with AT-RvD1 (p=0.0669) (**Figure 8C**). Finally, *il1b* was significantly increased in WT DE-exposed, AT-RvD1-treated mice compared to KO DE-exposed, AT-RvD1-treated mice (p=0.0228) (**Figure 8D**). In the frontal cortex, tgfb displayed a trend of increased transcription between WT and KO mice exposed to saline (p=0.0600), and a trend of reduced transcription between KO mice exposed to DE and KO mice exposed to saline (p=0.0560) (**Figure 8F**). Evaluation of the isocortex revealed significantly increased *tgfb* transcription between WT and KO saline-exposed mice (p=0. 0355), as well as significantly increased *tgfb* expression in KO mice exposed to DE compared to KO saline-exposed controls (p=0.0355) (**Figure 8J**). We also found that *il1b* expression was significantly reduced in WT DE-exposed, AT-RvD1 treated mice compared to KO DE-exposed, AT-RvD1 treated mice (p=0.0245) (**Figure 8L**). Transcript evaluation of the hippocampus revealed significantly increased *tgfb* expression in KO saline-exposed, saline-treated mice compared to WT saline-exposed, saline-treated mice (p=0.0049) (**Figure 8N**) and a significant decrease in *tgfb* expression in KO DE-exposed, saline-treated mice compared to KO saline-exposed, saline-treated controls (p=0.0198) (**Figure 8N**). We did not observe any significant differences or trends in the cerebellum. We did observe a change in the hindbrain, with *il1b* transcripts significantly decreased in WT saline-exposed and AT-RvD1 treated mice compared to WT saline-exposed, saline-treated (p=0.0252) (**Figure 8X**).

## Discussion

The pulmonary inflammatory response to ODE has been well established, however the neuroinflammatory effects of this environmental and occupational exposure are not clear (10, 16, 47). Other models of environmental exposures examining neuroinflammation have demonstrated that inhalant exposures can be associated with neuroinflammation and can ultimately lead to neurodegenerative disease (3, 22, 25, 58). In addition, a pulmonary infection model *of Mycobacterium tuberculosis* demonstrated severe pulmonary bacterial infection without microbial dissemination to the brain that produced gliosis, neuroinflammation, and misfolded proteins, pathologies of animals developing neurodegenerative disease (2). Epidemiological studies have also linked pulmonary inflammation and infection in patients with COPD and *Mycobacterium tuberculosis* to neurological disease and early signs of neurodegenerative disease including cognitive decline, depression and anxiety, reduced social interaction, and ultimately reduced quality of life (27, 28, 59). To assess whether ODE contributes to neurological inflammation, microglia cell numbers were evaluated as an indicator of inflammation (2, 60, 61). Glial cells such as microglia are the primary mediators of the neuroinflammatory response and have been evaluated as markers of neuroinflammation in other peripheral inflammation models (2, 59). Proliferation and migration of microglia in different brain regions have been implicated in neurodegenerative disease and impaired neuron functionality. Increases in the number of microglia indicate inflammation or inflammatory processes, while reduction of these cells indicates resolution of inflammation (55–57). AT-RvD1 has shown efficacy in reducing neuroinflammation, suggesting that it may be an effective treatment to restore microglia homeostasis in deficient models, and our data demonstrates that AT-RvD1 may be effective in reducing microglia numbers in a mouse model of ODE (15, 62).

We found that proliferation of microglia primarily occurred in the rostral brain areas assessed: the olfactory bulb, frontal cortex, and isocortex. This may be attributed to the route of DE exposure, intranasal instillation, and the proximity of these brain regions to the olfactory system. One study found that two intranasal instillations of carbon nanoparticles were disseminated into the olfactory bulbs, which may be a mechanism of the observed gliosis, given the localization of the gliosis (54). Another possible mechanism is that peripheral inflammation can contribute to gliosis without translocation of the inflammatory substances to the brain, as observed in a study examining gliosis in a guinea pig model of *Mycobacterium tuberculosis* infection observed gliosis without dissemination of bacteria to the brain (2). It is also possible that the components of the DE used in this study, such as lipopolysaccharides (LPS) could be transported to the brain through transport proteins and be directly activating microglia via TLR4 pathways (63). We however did not observe an increase in IL-1β transcripts in brains of mice exposed to DE, so further evaluation of the cytokines profile and signaling is needed. We hypothesize that the inflammatory response observed in the brain of animals exposed to DE is TLR-mediated by microglia, however more information is required to decern the pathways and mechanisms that contribute to neuroinflammation in organic dust exposure. Our study is limited by the intranasal instillation of DE technique that is an effectively sterile, non-live infection model that may not be directly translatable to occupation exposure to ODE, which contains larger particles and live microbes (14, 47, 62). In addition, our mice are also only exposed a total of 15 times, which may not provide a sufficient exposure duration to elicit a robust neurological inflammatory response in all brain regions. Our laboratory has previously explored the role of IL-22 in the pulmonary immune response to ODE, but we have not examined the role of this cytokine in a neurological inflammatory model or in a recovery model of repetitive ODE (45). We previously explored this knock-out model in a 15-intstillation repetitive exposure model, however this study examining tissues 5 hours post-last DE instillation, but we have not yet explored the recovery period in the days following cessation of DE exposure (45). We have also previously examined the therapeutic actions of AT-RvD1 in a chronic dust exposure model but have not explored its applications in an ODE-induced neuroinflammatory model (32). We investigated the neuroinflammatory response to agriculture dust, the role of IL-22 in pulmonary and neurological inflammation, and the therapeutic applications of AT-RvD1 in a pulmonary and neurological IL-22 knock-out mouse model repetitively exposed to agriculture dust extract and treated with AT-RvD1. We found that in many of our parameters, IL-22 KO mice displayed increased disease severity via increased iBALT percentage (**Figure 4B**), increased microglia numbers in the olfactory bulb and cerebellum of saline-exposed animals (**Figures 7B, J**), and many of the differences in expression of cytokines and mediators evaluated at the protein (**Figures 5D, E**) and transcript (**Figures 6B, F**, **8B, D, F, J, L, N, P**) levels were genotype-driven. We observed a significant increase in the total cells collected and lymphocyte differential counts in both WT and KO animals following DE exposure and a significant influx of macrophages in KO animals. This is consistent with our previous findings that IL-22 KO is associated with increased total and differential cell counts after DE exposure (**Figure 2A**) (45). However, we did find that the differential cell counts differed in our 72-hour recovery model. This is likely due to a shift in the immune response from an innate response characterized by high neutrophil counts to a more adaptive response dominated by lymphocytes (**Figures 2A–E**) (45, 64). KO animals treated with 250 ng AT-RvD1 once per week displayed decreased total cells and lymphocytes. KO animals also displayed a trend of increased total cell counts and significantly increased lymphocytes compared to WT animals. These data suggest that IL-22 knock-out may modulate cell recruitment to sites of injury and that AT-RvD1 also regulates immune cell recruitment, a well-known action of this SPM (34, 38, 43, 45, 65). In addition, KO animals displayed increased pathology severity including increased iBALT percentage in DE-exposed animals compared to WT DE-exposed animals (**Figure 4B**). This may also be due to altered lymphocytic recruitment as iBALT is largely composed of organized B and T cells (66–68).One inflammatory mediator, AREG is involved in modulating repair and remodeling in the lung after injury and has previously been evaluated in a repetitive 15-instillation ODE model where mice were allowed to recover for 1, 2, 3, or 4 weeks post-last DE instillation (50). However, the modulation of AREG has not been examined in a repetitive ODE model with a shorter, 72-hour recovery period or in an IL-22 knock-out model treated with AT-RvD1 (50). In our previous study, we observed that AREG concentrations in BALF increased as the recovery timepoints lengthened following cessation of DE exposure (50). Interestingly, we did not find that AREG was upregulated in WT animals exposed to DE, but we did observe that KO mice exposed to DE and recovered for 72 hours displayed significantly decreased AREG tissue concentrations compared to WT mice exposed to DE. Interestingly, KO mice administered AT-RvD1 also displayed a significant decrease in AREG lung tissue concentrations compared to WT mice exposed to DE and treated with AT-RvD1. This may suggest that IL-22 may play an important role in effective tissue repair and resolution response after DE exposure as other models have found that IL-22 knock-out results in reduced skin wound healing, however the specific mechanisms warrant further investigation (69, 70).

We previously explored the use of AT-RvD1 treatment in a chronic 24-week ODE mouse model and observed significantly reduced pathology severity in mice injected with 500 ng AT-RvD1 via intravenous tail injection once weekly for 20 weeks (32). We found that this dose was effective in reducing lung inflammation and cellular infiltration in this model and aimed to evaluate the efficacy of AT-RvD1 in reducing ODE-induced pulmonary and neurological inflammation in a repetitive 15-instillation exposure model (32). We evaluated the neurological immune response via microglia counts and mRNA transcript evaluation using RNAscope in several brain regions. We utilized two dosing regimen models to determine which strategy was most effective. We first examined cellular infiltrates and histopathology in animals treated with 250 ng AT-RvD1 once-weekly (**Figures 1A**, **2A–E**, **3A–D**) and found limited efficacy in immune cell influx, and no therapeutic efficacy in lung histopathology (**Figures 3A–D**). We then evaluated lung histopathology outcomes in a once-daily AT-RvD1 injection schedule and found that animals treated with 250 ng AT-RvD1 daily displayed significant inflammation resolution in histopathology evaluation (**Figures 4A–H**), therefore, our 5 day/week injection regimen was employed for further analysis of the pulmonary and neurological inflammatory response to DE. Our histopathological analysis of animals administered daily AT-RvD1 injections revealed that iBALT was the primary parameter improved in AT-RvD1-treated animals. iBALT is considered a pathological phenotype of COPD that is correlated with disease severity and its increased formation has been linked to increased severity of patient clinical signs (71–73). We have previously found that mice exposed to DE, whether repetitively or chronically, develop significant iBALT (32, 45, 50, 51). In this study, we observed that WT and KO mice exposed to DE revealed increased iBALT formation, with significantly increased iBALT percentage in female mice compared to male mice. This may be due to sex hormones contributing to adaptive immunity to varying degrees, however our study is limited by a lack of estrus cycling and sex hormone quantification data that would be needed to make more informed conclusions on the mechanisms of the observed sex-specific pathology (74). Furthermore, female animals treated with AT-RvD1 displayed significantly less iBALT formation, indicating its effectiveness in reducing immune cell aggregates in these animals, but more investigation into the mechanism of these sex differences is warranted. This pattern of increased lung pathology severity in females has been documented in human patients with COPD, where female patients tend to experience more severe symptoms and have higher mortality rates (54, 59). Together, these data demonstrate that attenuation of IL-22 alters the pulmonary immune response to agriculture dust and increases the severity of lung pathology and that the efficacy of AT-RvD1 attenuation of lung pathology is IL-22- and sex-dependent. Females have also demonstrated higher toll-like receptor production, present with more CD4^+^ T cells and B cells compared to males and have a more robust antibody response than males (74). The increased T and B cell numbers may account for the reduced resolution of iBALT in females, but more investigation of the mechanisms of these sex differences in our dust exposure model is warranted.

mRNA transcript evaluation of whole lung tissue sections revealed significant differences in *cxcl10* expression in WT mice exposed to DE with a significant decrease in WT animals exposed to DE and treated with AT-RvD1. CXCL10 participates in monocyte, neutrophil, and lymphocyte recruitment and significantly contributes to the progression of COPD disease state, which may present as a therapeutic target of AT-RvD1 in dust-induced lung disease (21, 75, 76). mRNA transcript evaluation of brains revealed limited significant differences between treatment groups, with significantly reduced *il1b* expression in WT animals exposed to DE and treated with AT-RvD1 compared to KO animals exposed to DE and treated with AT-RvD1. The mechanism of this observation is unclear, but another study found that cultured microglia displayed reduced *il1b* expression after treatment with 17(S)-RvD1 (77). This is not directly comparable to our model of AT-RvD1 (17(R)-RvD1) treatment but may still be informative as we explore the therapeutic targets of AT-RvD1. A major limitation of this study is our restricted panel of mRNA transcripts in both the lung and brain tissues, with only one pro-inflammatory mediator included per tissue type. This has hindered our investigation of the therapeutic applications of AT-RvD1, and we may simply be missing those mediators that are affected by At-RvD1 treatment. Another limitation is our 72-hour post-last instillation timepoint, where the mice are primarily in the resolution phase and may not be producing high quantities of pro-inflammatory cytokines. Expansion of our investigation of mRNA transcripts to include a broader range of classic inflammatory mediators may assist our assessment of the contribution of ODE to neuroinflammation and the therapeutic actions of AT-RvD1 in our ODE model.

We aimed to determine whether agriculture dust exposure contributes to neurological inflammation in a repetitive ODE mouse model. Our data support the hypothesis that agriculture dust exposure contributes microgliosis in a mouse model of repetitive ODE. We also aimed to assess the regulation of the pulmonary and neurological inflammatory response to ODE in an IL-22 deficient mouse model and the immune-modulating and therapeutic applications of AT-RvD1. Our data show that ODE leads to pulmonary and neurological inflammation, and that IL-22 attenuation increases the severity of the pulmonary and neurological immune response to ODE. This study confirmed our previous findings and expanded upon our prior knowledge by demonstrating that IL-22 KO mice exhibit increased inflammatory markers in the recovery phase following cessation of DE exposure. We also demonstrate that AT-RvD1 treatment once per week was effective in reducing cellular infiltrates in BALF of KO animals, but did not produce a significant decrease in lung pathology outcomes. We observed that a once daily regimen was more effective in increasing lung anti-inflammatory cytokine protein production, reducing lung pathology severity, reducing lung chemokine transcript expression, and decreasing microglia numbers in mice exposed to DE. These data indicate that AT-RvD1 may require more frequent administration to be effective, however more evidence and exploration is needed. We also demonstrated that AT-RvD1 treatment is more effective in IL-22 KO mice, in terms of reducing lung cellular infiltrates and pro-inflammatory cytokine protein concentrations, but that it is also effective in reducing lung pathology and microglia numbers in both WT and KO animals. We hypothesize that this is due to a dysregulation of the repair processes in KO mice, which is partially restored by AT-RvD1 treatment, but requires further investigation to determine the specific pathways and mechanisms involved in these observations.

Further investigation aims to identify the mechanisms by which ODE contributes to neurological inflammation through evaluation of various routes of escape from the pulmonary to the neurological systems and the specific cellular mechanisms that lead to neuroinflammation in a model of ODE. These data demonstrate that ODE leads to neurological inflammation and that AT-RvD1 may be an effective treatment for attenuating immune-mediated pulmonary and neurological disease caused by ODE, mitigating its severe health effects.

## Data Availability

The raw data supporting the conclusions of this article will be made available by the authors, without undue reservation.
